# A first step in determining appropriate amounts of obstetric anesthesia work

**DOI:** 10.1186/2045-4015-1-49

**Published:** 2012-12-14

**Authors:** Swarup S Varaday, Barbara L Leighton

**Affiliations:** 1 Anesthesiology, Washington University School of Medicine, Campus Box 8054, 660 South Euclid Avenue, Saint Louis, Missouri, 63110, USA; 2 Anesthesiology and Obstetrics and Gynecology, Washington University School of Medicine, Campus Box 8054, 660 South Euclid Avenue, Saint Louis, Missouri, 63110, USA

**Keywords:** Obstetric anesthesia, Manpower, Workload

## Abstract

Ginosar, *et al.* describe a new performance indicator, the Obstetric Anesthesia Activity Index, to represent the current amount of obstetric anesthesia work done daily at each of 25 Israeli hospitals. The authors claim, correctly, that this index is a closer reflection of the anesthetic workload than simply looking at the number of deliveries at each hospital. However, the Obstetric Anesthesia Activity Index could easily be refined to reflect more closely the actual obstetric anesthesia workload by using the average cesarean delivery time for each hospital rather than one value for all hospitals. Although the authors state that they developed the Obstetric Anesthesia Activity Index out of concern for inadequate obstetric anesthesia manpower in Israel, they have not compared the Obstetric Anesthesia Activity Index with the size of the patient population or any measure of patient satisfaction or patient safety. In its current form, the Obstetric Anesthesia Activity Index describes the current work situation but does not evaluate the extent of the unmet need for additional anesthesia providers. Despite these shortcomings, the Obstetric Anesthesia Activity Index is an important first step in developing a tool to assess unmet obstetric anesthesia needs.

## Background

There is currently no generally accepted way to compare the amount of obstetric work performed at different hospitals. Israel is currently facing an anesthesia manpower shortage, and the authors developed the Obstetric Anesthesia Activity Index to measure the amount of obstetric anesthesia work performed at each hospital in Israel.

## Discussion

In their article “Comparison of the obstetric anesthesia activity index with total delivery numbers as a single denominator of workload demand in Israeli maternity units”, Dr. Ginosar and his coauthors present a new performance indicator, the Obstetric Anesthesia Activity Index (OAAI), to represent the current amount of obstetric anesthesia work done daily at each of 25 Israeli hospitals [1]. The OAAI is an extension and improvement on the obstetric anesthesia workload measure developed by Yentis and Robinson [2].

Ginosar, *et al*., claim, correctly, that this index is a closer reflection of the anesthetic workload than simply looking at the number of deliveries at each hospital. However, the OAAI could easily be refined to reflect more closely the actual obstetric anesthesia workload in each hospital. The authors assume that the average time for epidural placement in each hospital is 45 minutes and the average time of cesarean delivery is 90 minutes. The actual average times probably vary greatly among hospitals, especially between university and private hospitals. For example, our division provides obstetric anesthesia care at a university hospital, where the average cesarean time is 98 minutes, and a private hospital, where the average cesarean time is 73 minutes. In many countries, exact times can usually be obtained from billing data or electronic anesthesia records.

The authors claim that OAAI is a dimensionless number. This is problematic and confusing; OAAI is the average number of hours per day the obstetric anesthesia team spends initiating regional anesthesia for labor and performing anesthesia for cesarean delivery. As the authors themselves acknowledge, OAAI is not a measure of the total activity of the obstetric anesthesia services. The ideal measure of anesthetic activity would include (for all indications): all antepartum assessments and educational classes; all intrapartum attendances for anesthetic advice, monitoring, etc.; all regional blocks instituted; all subsequent visits including top-ups, resisting and adjustments to catheters; all cesarean deliveries; and all postoperative and postpartum follow-up, postoperative pain-relief and general care. However, the OAAI does include the two most time-consuming parts of the obstetric anesthesia workload and is thus a valid way to compare different hospitals.

The authors correctly state that OAAI does not consider the degree of workforce redundancy that is required to safely accommodate extra workload during peak activity or provide expert back-up when the maternity services are located in remote locations away from the main anesthesia services, especially emergency cesareans. Also, the data could not differentiate elective from emergency cesareans.

Although the authors state that they developed the OAAI out of concern for inadequate obstetric anesthesia manpower in Israel, the authors have not compared OAAI with the size of the patient population or any measure of patient satisfaction or patient safety. An index including the amount of obstetric anesthesia work performed relative to the number of hospital deliveries should be correlated with the time patients wait after requesting labor epidurals, the wait time for unscheduled cesarean deliveries, or with the percent of patients who delivered before an anesthesiologist was available to place a labor epidural. In its current form, OAAI describes the current work situation but does not evaluate the extent of the unmet need for additional anesthesia providers. Studies have been done in other fields to evaluate the effect of work force differences on the quality of patient care [3].

## Conclusions

The Obstetric Anesthesia Activity Index combines the time spent placing labor epidurals and performing cesarean deliveries to generate a measure of the total amount of obstetric anesthesia work performed at a given hospital. The index would be improved if average times for cesarean delivery for each hospital were incorporated in the formula, rather than assuming a constant average time at all hospitals. The authors have not correlated the index with any patient safety or satisfaction outcomes or with delivery numbers, so there is no way to know if the amount of obstetric work performed is appropriate for the size of the patient population. Despite these shortcomings, the Obstetric Anesthesia Activity Index is an important first step in developing a tool to assess unmet obstetric anesthesia needs. We hope that the authors will extend their work, for it is unlikely that needs that are not defined will be addressed.

## Competing interests

The authors declare that they have no competing interests.

## Authors’ contributions

SSV, MBBS FRCA FCARSI, completed his medical school and anesthesia training in India. He attended Imperial College, London for further anesthesia training and worked as a consultant in Anesthesia and Intensive care in the United Kingdom. He is currently Assistant Professor of Anesthesiology at Washington University in St. Louis and is the perioperative department co-leader for minimally invasive surgery and obstetric anesthesia. BLL, MD, graduated from the Johns Hopkins University School of Medicine and completed her anesthesiology residency and obstetric anesthesiology fellowship at the University of Pennsylvania. She is currently a Professor of Anesthesiology and Obstetrics and Gynecology and is the Chief of the Section of Obstetric Anesthesiology at Washington University in St. Louis.

## Authors’ information

Commentary on the paper by Y. Ginosar, *et al*. Comparison of the obstetric anesthesia activity index with total delivery numbers as a single denominator of workload demand in Israeli maternity units.
